# Supplementary material for: miR-140-5p Attenuates Hypoxia-Induced Breast Cancer Progression by Targeting Nrf2/HO-1 Axis in a Keap1-Independent Mechanism
Source: Cells. 2021 Dec 22;11(1):12. doi: 10.3390/cells11010012 (PMC8750786; doi:10.3390/cells11010012)
Supplement: Supplementary file 1 [file cells-11-00012-s001.zip › cells-1418549-supplementary.pdf]

miR-140-5p Attenuates Hypoxia-Induced Breast Cancer
Progression by Targeting Nrf2/HO-1 Axis in a Keap1-Independent Mechanism

Megharani Mahajan and **Sandhya Sitasawad** *

Redox Biology Lab, National Centre for Cell Science (NCCS), Pune 411007, India;
mmegha@nccs.res.in or meghanandgaon@gmail.com
* Correspondence: ssitaswad@nccs.res.in; Tel.: +20-25-708-148

Supplementary materials and methods

Antibody staining by FACS

The Hif-1α level was determined by flow cytometry as described previously (1).

Transfection

Transient transfection of the shHif-1α vector was performed as reported previously (2).

Adhesion assay

Cell-ECM (Extracellular matrix adhesion) was analyzed by performing adhesion assay as reported previously (3).

Cell cycle analysis

Cell cycle analysis was performed using flow cytometry as described previously (4).

References

1. Dasgupta, Aparajita, et al. "AECHL-1 targets breast cancer progression via inhibition of metastasis, prevention of EMT and suppression of Cancer Stem Cell characteristics." Scientific reports 6.1 (2016): 1-13, https://doi.org/10.1038/srep38045.
2. Tian, Nianxiu, et al. "Emodin mitigates podocytes apoptosis induced by endoplasmic reticulum stress through the inhibition of the PERK pathway in diabetic nephropathy." Drug Design, Development and Therapy 12 (2018): 2195, https://doi.org/10.2147/dddt.s167405.
3. Penna, Elisa, et al. "microRNA-214 contributes to melanoma tumour progression through suppression of TFAP2C." The EMBO journal 30.10 (2011): 1990-2007, https://doi.org/10.1038/emboj.2011.102.
4. Yu, Junhui, et al. "MicroRNA-181a promotes cell proliferation and inhibits apoptosis in gastric cancer by targeting RASSF1A." Oncology reports 40.4 (2018): 1959-1970, https://doi.org/10.3892/or.2018.6632.

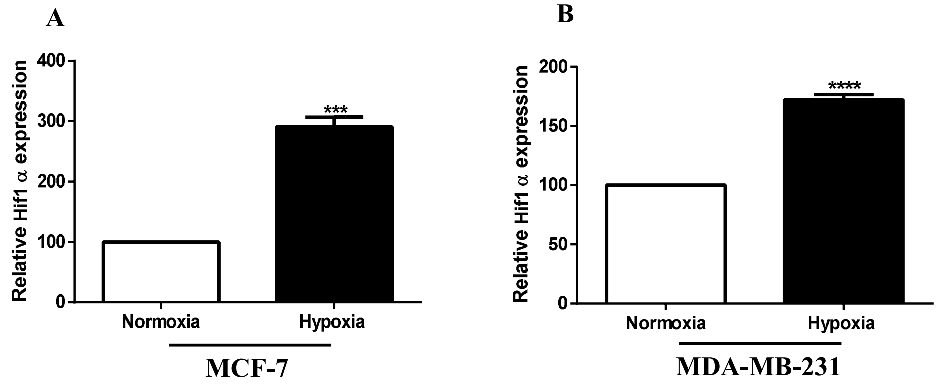

Figure S1. Hypoxia induces a change in Hif-1α protein level.

BC cells were exposed to hypoxia for 48 h. Hif1α level was measured by flow cytometry in MCF-7 (A) and MDA-MB-231 (B). Results were represented as relative fluorescence intensity. Error bars indicate mean ± SEM (n = 3). Student's t-tests were used to compare the means of two groups. ***P < 0.001 and ****P<0.0001 compared to Normoxia.

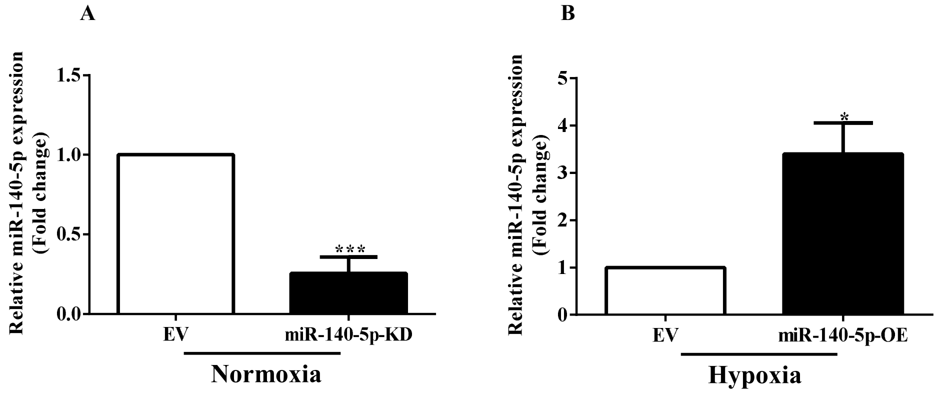

Figure S2. Confirmation of miR-140-5p knockdown and overexpression. MDA-MB-231 cells were transduced with lentivirus containing miR-140-5p knockdown or overexpression constructs. The efficiency of miR-140-5p knockdown under normoxia (A) or overexpression under hypoxia (B) was analyzed by measuring its relative expression by qRT-PCR. Error bars indicate mean ± SEM (n = 3). Student's t-tests were used to compare the means of two groups. *P<0.05 and ***P < 0.001 compared to EV.

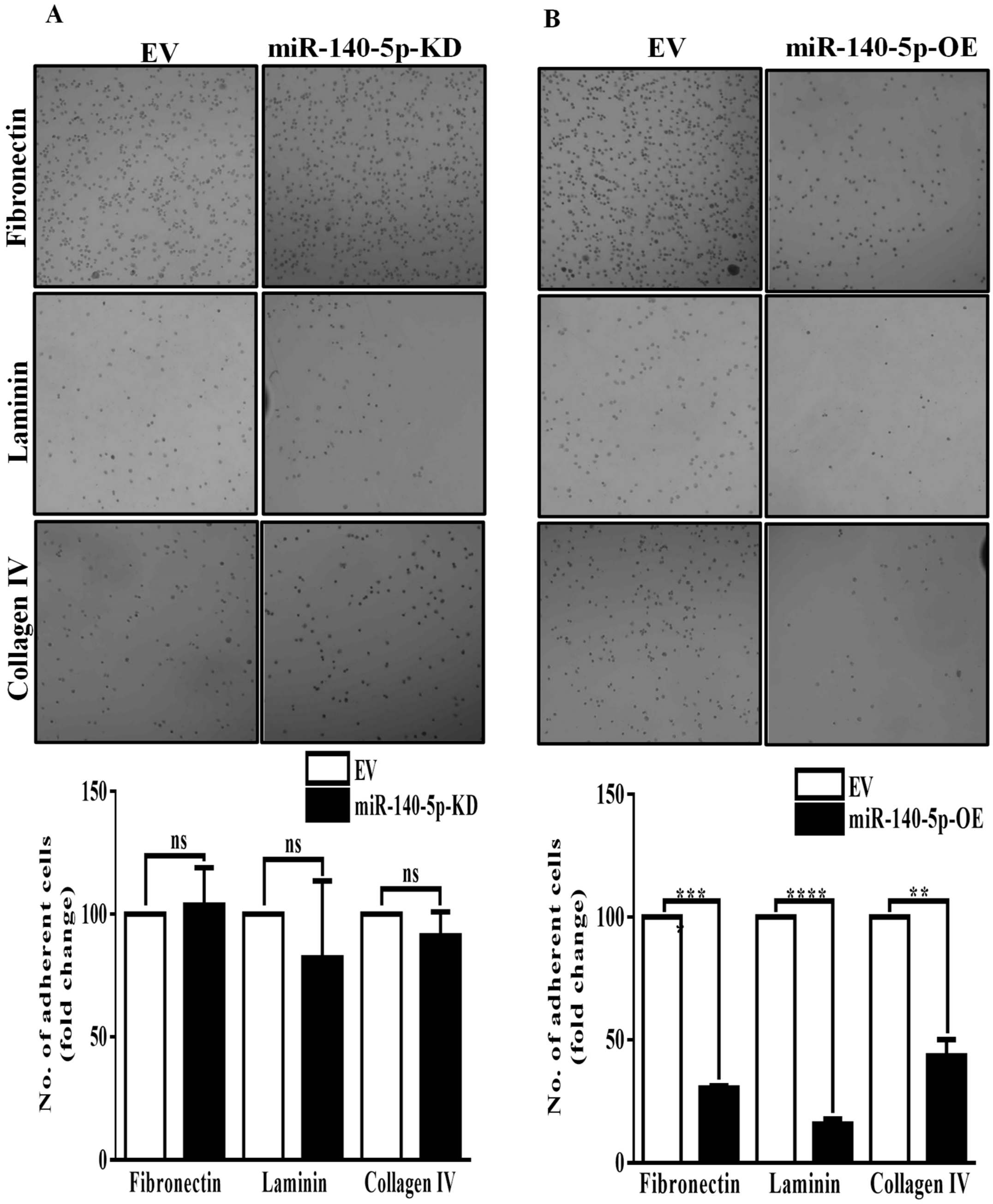

Figure S3. miR-140-5p modulates cell adhesion. Cell adhesion assay of MDA-MB-231 cells adhering to Fibronectin, Laminin, or Collagen IV. (A) miR-140-5p knockdown under normoxia or (B) miR-140-5p overexpression under hypoxia along with their EV control. Error bars indicate mean ± SEM (n = 3). Student's t-tests were used to compare the means of two groups. ns: not significant, **P<0.01, ***P < 0.001 and ****P<0.0001 compared to EV.

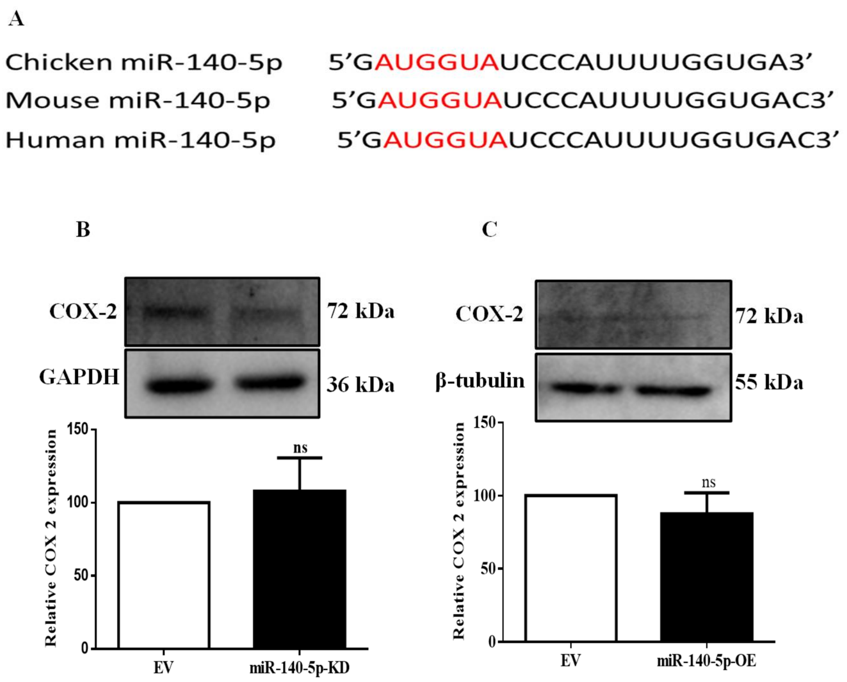

Figure S4. Effect of miR-140-5p expression on angiogenic marker. (A) Schematic representation of conserved seed sequence of miR-140-5p across different species. (B) Representative western blot images and its densitometry of COX-2, showing the non-significant change in the level in miR-140-5p knockdown under normoxia (B) or overexpression under hypoxia (C). GAPDH and β-tubulin were used as endogenous controls. Error bars indicate mean ± SEM (n = 3). Student's t-tests were used to compare the means of two groups. ns: not significant.

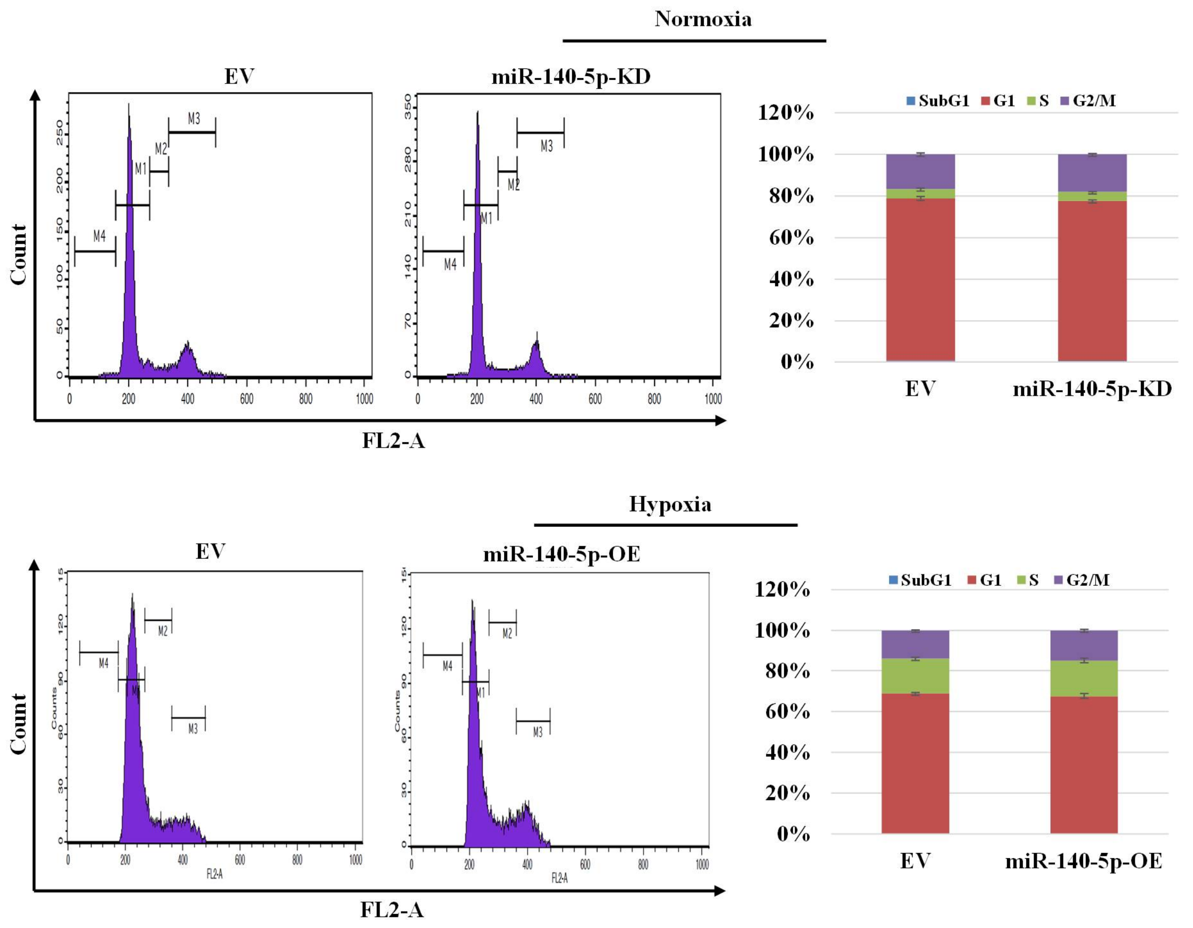

Figure S5. Effect of miR-140-5p expression on cell cycle. Effect of miR-140-5p expression on different cell cycle phases in MDA-MB-231 cells with stable miR-140-5p-KD under normoxia (A) or miR-140-5p-OE under hypoxia (B) were assessed by flow cytometry. Representative graphs from three independent experiments were shown. Error bars indicate mean ± SEM (n = 3).

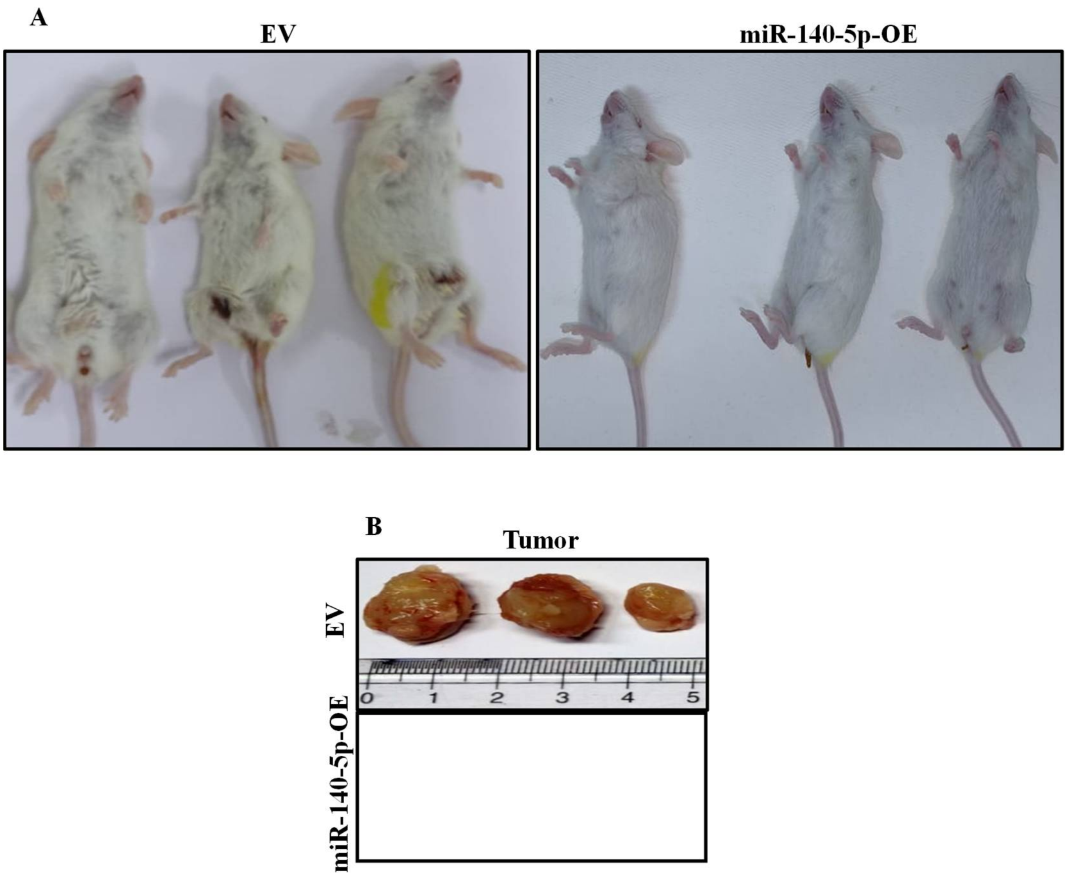

Figure S6. Tumorigenicity assay in mouse xenograft model. (A) MDA-MB-231 cells with EV or miR-140-5p-OE were subcutaneously injected into the mammary fat pad of female SCID mice. (B) Representative image of tumors from EV group. Since tumor growth was potently suppressed, mice in the miR-140-5p-OE group did not develop any tumor. n=3.

Table S1. Prediction of novel miRNAs targeting Nrf2 mRNA

Target Scan	miRDB	miRSystem
hsa-miR-27-3p	hsa-miR-144-3p	hsa-miR-101-3p
hsa-miR-128-3p	hsa-miR-3680-3p	hsa-miR-106b-5p
hsa-miR-142-5p	hsa-miR-582-5p	hsa-miR-128-3p
hsa-miR-153-3p	hsa-miR-212-3p	hsa-miR-129-5p
hsa-miR-144-3p	hsa-miR-132-3p	hsa-miR-132-3p
hsa-miR-140-5p	hsa-miR-6854-5p	hsa-miR-140-5p
	hsa-miR-340-5p	hsa-miR-142-3p
	hsa-miR-153-3p	hsa-miR-142-5p
	hsa-miR-450b-5p	hsa-miR-144-3p
	hsa-miR-500a-5p	hsa-miR-153-3p
	hsa-miR-5590-3p	hsa-miR-186-5p
	hsa-miR-7-1-3p	hsa-miR-199a-3p
	hsa-miR-142-5p	hsa-miR-199b-3p
	hsa-miR-140-5p	hsa-miR-20a-5p
	hsa-miR-7-2-3p	hsa-miR-20b-5p
		hsa-miR-212-3p
		hsa-miR-27a-3p
		hsa-miR-27b-3p
		hsa-miR-28-5p

Table S2. Primer sequences used for generation of vector and qPCR

Gene name	Primer orientation	Sequence (5’→3')
miR-142-5p	Stem loop	GTCGTATCCAGTGCAGGGTCCGAGGTATTCGCACTGGATACGACAGTAGT
miR-142-5p	Forward	CCATAAAGTAGAAAGCACTAC
miR-153-3p SL	Stem loop	GTCGTATCCAGTGCAGGGTCCGAGGTATTCGCACTGGATACGACGATCAC
miR-153-3p	Forward	CTTGCATAGTCACAAAAGTGA
miR-144-3p SL	Stem loop	GTCGTATCCAGTGCAGGGTCCGAGGTATTCGCACTGGATACGACAGTACA
miR-144-3p	Forward	CTACAGTATAGATGATGTACT
miR-140-5p	Stem loop	GTCGTATCCAGTGCAGGGTCCGAGGTATTCGCACTGGATACGACCTACCA
miR-140-5p	Forward	GTATACCAGTGGTTTTACCCT
miRNA	U/R	CCAGTGCAGGGTCCGAGGTA
U6	Forward	CTCGCTTCGGCAGCACA
U6	Reverse	AACGCTTCACGAATTTGCGT
Nrf2	Forward	GAGAGCCCAGTCTTCATTGC
Nrf2	Reverse	TGCTCAATGTCCTGTTGCAT
HO-1	Forward	CTGGAGGAGGAGATTGAGCG
HO-1	Reverse	ATGGCTGGTGTGTAGGGGAT
Keap1	Forward	TACGATGTGGAAACAGAGACGTGGACTTTCGTA
Keap1	Reverse	TCAACAGGTACAGTTCTGGTCAATCTGCTT
Twist	Forward	GCCAGGTACATCGACTTCCTCT
Twist	Reverse	TCCATCCTCCAGACCGAGAAGG
Snail	Forward	GCTGCAGGACTCTAATCCAGA
Snail	Reverse	ATCTCCGGAGGTGGGATG
Slug	Forward	TGGTTGCTTCAAGGACACAT
Slug	Reverse	GTTGCAGTGAGGGCAAGAA
Vimentin	Forward	AGGCAAAGCAGGAGTCCACTGA
Vimentin	Reverse	ATCTGGCGTTCCAGGGACTCAT
β-catenin	Forward	CACAAGCAGAGTGCTGAAGGTG
β-catenin	Reverse	GATTCCTGAGAGTCCAAAGACAG

GAPDH	Forward	CCTGCACCACCAACTGCTTAG
GAPDH	Reverse	TGAGTCCTTCCACGATACCAA
Nrf2 3'UTR (WT)	Forward	TCGAGTAAAAAGAAATTATTGCAAAACTAACCACTATGTACTTTTTTATAAATGC
Nrf2 3'UTR (WT)	Reverse	GGCCGCATTTATAAAAAAGTACATAGTGGTTAGTTTTGCAATAATTTCTTTTTAC
Nrf2 3'UTR (MUT)	Forward	TCGAGTAAAAAGAAATTATTGCAAAACTATCGATTCTGTACTTTTTTATAAATGC
Nrf2 3'UTR (MUT)	Reverse	GGCCGCATTTATAAAAAAGTACAGAATCGATAGTTTTGCAATAATTTCTTTTTAC
miR-140-5p (o/e)	Forward	CCGGCTACCATAGGGTAAAACCACTGCTCGAGCAGTGGTTTTACCCTATGGTAGTTTTTG
miR-140-5p (o/e)	Reverse	AATTCAAAAACTACCATAGGGTAAAACCACTGCTCGAGCAGTGGTTTTACCCTATGGTAG
TuD-NC	Forward	5’CCGGTGACGGCGCTAGGATCATCAACAAGCCACAACGAATCTCTATATCATCAAGTATTCTGGTCACAGAATACAACAAGCCACAACGAATCTCTATATCATCAAGATGATCCTAGCGCCGTCTTTTTTG-3’
TuD-NC	Reverse	5’AATTCAAAAAAGACGGCGCTAGGATCATCTTGATGATATAGAGATTCGTTGTGGCTTGTTGTATTCTGTGACCAGAATACTTGATGATATAGAGATTCGTTGTGGCTTGTTGATGATCCTAGCGCCGTCA -3’
TuD miR-140-5p	Forward	5’CCGGTGACGGCGCTAGGATCATCAACCTACCATAGGGTATCTAAAACCACTGCAAGTATTCTGGTCACAGAATACAACCTACCATAGGGTATCTAAAACCACTGCAAGATGATCCTAGCGCCGTCTTTTTTG3’
TuD miR-140-5p	Reverse	5’AATTCAAAAAAGACGGCGCTAGGATCATCTTGCAGTGGTTTTAGATACCCTATGGTAGGTTGTATTCTGTGACCAGAATACTTGCAGTGGTTTTAGATACCCTATGGTAGGTTGATGATCCTAGCGCCGTCA3
shHif1α	Forward	CCGGCCAGTTATGATTGTGAAGTTACTCGAGTAACTTCACAATCATAACTGGTTTTTG
shHif1α	Reverse	AATTCAAAAACCAGTTATGATTGTGAAGTTACTCGAGTAACTTCACAATCATAACTGG

Table S3. List of antibodies

**Antibody**	**Catalogue no.**	**Company**	**Species**
Hif1α	sc-13515	Santa Cruz	Mouse
Nrf2	sc-722	Santa Cruz	Rabbit
Keap1	#8047S	CST	Rabbit
HO-1	5853S	CST	Rabbit
PCNA	sc-25280	Santa Cruz	Mouse
AKT1	Sc-5298	Santa Cruz	Mouse
pAKT1/2/3	sc-16646-R	Santa Cruz	Rabbit
ERK1	sc-94	Santa Cruz	Rabbit
pERK1/2	sc-7383	Santa Cruz	Mouse
VEGF	sc-7269	Santa Cruz	Mouse
COX-2	Sab2500267	Sigma	Goat
Vimentin	AB1620	Merck Millipore	Goat
Caspase3	#9668	CST	Mouse
Caspase7	#9492	CST	Rabbit
Caspase9	#9508	CST	Mouse
PARP	#9542	CST	Rabbit
BID	sc-11423	Santa Cruz	Rabbit
BAX	sc-493	Santa Cruz	Rabbit
Bcl2	sc-7382	Santa Cruz	Mouse
Slug	ab-27568	Abcam	Rabbit
Twist	T6451	Sigma	Rabbit

β-catenin	sc-7963	Santa Cruz	Mouse
E-cadherin	610182	BD Biosciences	Mouse
β-tubulin	T8328	Sigma	Mouse
GAPDH	G9545	Sigma	Rabbit
